# The impact of procalcitonin in assessing outcomes in pediatrics severe trauma cases: A three-year experience from a tertiary hospital

**DOI:** 10.37796/2211-8039.1388

**Published:** 2023-03-01

**Authors:** Waleed H. Albuali

**Affiliations:** Department of Pediatrics, College of Medicine, King Fahad Hospital of the University, Imam Abdulrahman Bin Faisal University, Dammam, Saudi Arabia

**Keywords:** Procalcitonin, Mortality, Pediatric intensive care unit, Ventilation

## Abstract

Summary

**Background:**

Although procalcitonin levels are raised in patients with systemic inflammation, its usage in pediatric patients, particularly those in the intensive care unit who are most susceptible to sepsis.

**Methods:**

It is a retrospective research study that included pediatric patients aged more than two weeks who were brought to the King Fahd Hospital of the University’s PICU owing to serious trauma or post-acute postoperative occurrences from January 2017 to December 2019. At 24 h after admission, data such as age, gender, comorbidities, trauma severity as measured by the Injury Severity Score, and PRISM III score were collected.

**Results:**

Following a surgery abscess, there were a total of 39 (15.9%) deaths. Patients who died during their hospital stay had significantly higher mean levels of biomarkers such as PRISM III, PCT at 24 h, PCT 48–72 h, and PCT at day 5 (p = 0.001). The area under the ROC curve for PCT level 48/72 h was 0.89 (% CI: 0.85–0.93), p = 0.001, indicating that PCT had highly significant predictive validity in predicting in-hospital mortality at the best cutoff point of >1.35 with a high level of accuracy and precision of 82.1% and 82.0%, respectively.

**Conclusion:**

The serum procalcitonin level (PTCL) can help predict the in-hospital prognosis of pediatrics that has had surgery. A combined control system is designed based on PTC expression for the examination of a patient receiving medication over a longer length of time.

## 1. Introduction

Despite ongoing advances in surgical equipment cleaning and good personal hygiene to undertake exploratory care and management operations in countries including Saudi Arabia [1,2], the rate of death of post-surgical widespread inflammatory disease syndrome (SIRS) in children keeps rising. The accessibility of quick, accurate, and particular tests is highly desired by doctors in the intensive care unit (ICU) for detecting infected patients and to prevent patients from the advantage of immediate basic antibiotic therapy [3–5]. Procalcitonin (PCT) is a peptide precursor of the hormone calcitonin. It is produced by the thyroid glands Para follicular cells and is involved in calcium homeostasis. Infectious stimuli, fungal infections, trauma and surgery can cause increases in serum PCT concentrations [6]. The use of biomarkers and prognostic scoring to detect infection, make a diagnosis, determine the severity of disease, and assess a patient’s outcome is a unique method. C-reactive protein (CRP) and taking longer to build PCT levels are currently the most commonly utilized markers in clinical settings. When compared to C-reactive protein (CRP) and leucocyte, correlation mortality with PCT level at a cut-off value of 6.38 ng/dl has the highest sensitivity and accuracy (81.8% and 80.8%, respectively, ROC area = 0.838), but the classification effectiveness of PCT in designed to detect acute infectious diseases and the severity of outcomes of sensitivity and selectivity on specific cut-off points still needs to be standardized [6]. Some other research of 105 neurotrauma cases [7] found that procalcitonin had an area under the ROC at entry of 0.76 (p = 0.05), which was considerably better than CRP, with an area under the ROC of 0.73 (p = 0.05). In a hospital-based study in Saudi Arabia, the overall rate of sepsis in ICU admitted patients was 16%, and SIRS was 8.1%, with a 40.3% fatality rate related to sepsis at Qassim University [8]. However, no study has reported the predictive effectiveness of PCT level in post-surgical sepsis as well as mortality rates in pediatric patients, with the exception of a new study in almost the same setting in Saudi Arabia done by Waleed B., et al. [9], which reported five years’ knowledge of diagnostic mortality using the procalcitonin level method for detection based on 400 patients who were admitted to PICU on mechanical ventilator. The current study took a step forward in looking into the use of procalcitonin as a biomarker in predicting mortality in pediatric patients who had an acute traumatic or post-surgical infection. An increased prognostic value using PCT mostly in assessment of in-hospital survival based on three main period of post PICU records provide some acceptable information to adopt procalcitonin assays as a basic usual protocol.

## 2. Methods

This was an observational longitudinal study based on an age group of acutely injured pediatric patients from our previous test subjects [9] who were admitted to the pediatric intensive care unit (PICU) of King Fahd Hospital of the University (KFHU) between January 2017 and December 2019 to determine the predictive validity of diagnostic markers in estimating in-hospital results of existence or risk of death. Prior to the start of the trial, approval from the hospital’s internal review board (IRB number: 2021-01-078) was acquired. It covers patients with either sex, aged 2 weeks to fourteen years, who are hospitalized in the PICU due to severe trauma or post-acute surgical events and also meet at least two of four characteristics, one of which seems to be abnormal temperatures or leukocyte total number: A temperature of 38.5 °C or less than 36 °C, 2) tachycardia, which is considered as a heart rate >2 SD above the median age, or bradycardia, which is defined as just a pulse rate below the 10th percentile. 3) a respiratory rate greater than two standard deviations above the age-related average, system for such an acute injury that isn’t caused by a neuromuscular disorder or the use of general anesthesia. 4) Absent chemotherapy-induced leukopenia, a high or low leukocyte count in relation to age, or a neutrophil count of more than 10% immature neutrophils patients who were admitted for less than 24 h, those who died within the first 24 h, those who had a cardiac arrest prior to PICU admission, those referred to the PICU because of injuries or chemical exposure, and those with missing or incorrect data have all been removed from this study. All clinical and analytical information was recorded. The ISS was used to classify the kind and intensity of trauma. Biomarker accuracy in the estimate of in-hospital outcome was assessed. Age, sex, comorbidity, the consequences of trauma according to Injury Scores or pediatric risk or mortality III (PRISM III) score at 24 h and PCT level at 24 h, 48 h, 72 h, and 5 days of admission, as well as blood culture, PICU stay, multi-organ failure, number of inotropes, and intubation need were all retrieved as necessary requirements. SPSS (version 20.0, IBM, Chicago, USA) was being used to analyze the data. Frequencies and percentages were used to depict all the categorical data. The Chi-square test, or Fisher’s exact test, was utilized for proportional comparisons in connection to deaths versus surviving consequences. The data was given as mean SD depending on age, weight, PICU duration, ventilator remaining, and marker levels. An unpaired t-test in the case of normally distributed data or a non-parametric Wilcoxon Mann–Whitney U-test in the case of non-Gaussian distribution was used for comparisons of random variables between expired and survived outcomes. The accuracy of the PRISM-III and PCT rates at 24 h, 48 h, 72 h, and five days following admission was determined using a ROC analysis. The likelihood of survival in relation to the length of stay in the PICU and also the usage of a mechanical ventilator was determined using KaplanSurvival Meier’s analysis. The P-value was set at 0.05, which would be statistically relevant.

## 3. Results

**Fig. 1:**
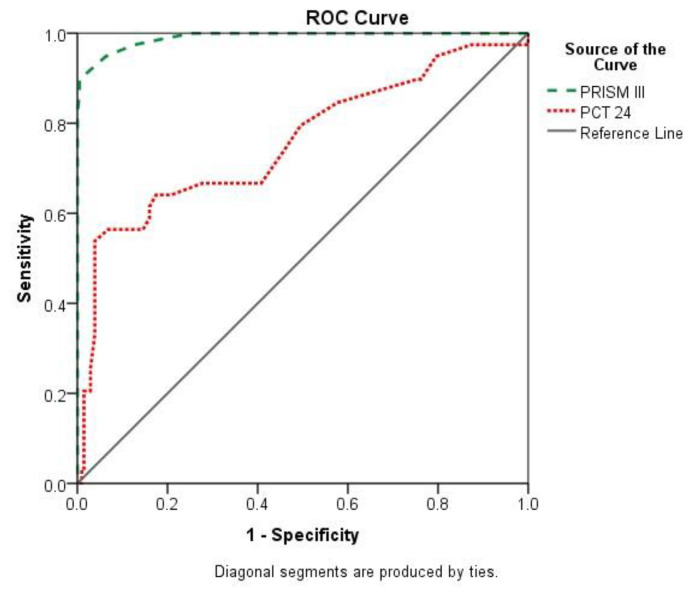
Comparison of predictive validity of PRISIM and PCT on 24 h, 48–72 h and PCT at day-5 to estimate patient’s in-hospital poor outcome. PRISM III score AUC = 0.99 (95% CI: 0.98–1.0), p < 0.001 (High predictive validity). PCT 24 h AUC = 0.76 (95% CI: 0.66–0.85), p < 0.001 (High predictive validity).

A total of 245 pediatric patients passed the study’s entry criteria, with an average age of 5.3 years and a male-to-female ratio of 58.8%. In 76 (31.0%) of the patients, sepsis was discovered. After surgical infection, there seemed to be a total of 39 (15.9%) deaths, with 22/39 (56.4%) of these being confirmed as having sepsis. As shown in Table 1, the pediatric patients’ in-hospital outcomes were non-significant when it came to gender (p = 0.461) and the necessity for a ventilator (p = 0.371), but they were highly significant when it came to type of origin, blood culture positive, and comorbidities (p < 0.001). Patients who died during their hospital stay had significantly higher mean rates of indicators such as PRISM III, PCT at 24 h, PCT 48–72 h, and PCT at day 5 (p < 0.001). As shown in Table 2, the passed group had considerably longer PICU stays, ventilation stays, and more vasopressor medicines than just the surviving group (p = 0.001), with the exception of mean age and weight, which were considerably lower in the diet group (p = 0.05). The receiver operating characteristic (ROC) curves and area under the curve (AUC) for the PRISM III score has been 0.99 (% CI: 0.98–1.00), p0.001, indicating that perhaps the PRISM III score had significant positive predictive value in the assessment of correlation morbidity at the greatest cutoff point of >9.5, with awareness, specificity, positive predictive value (PPV), and negative predictive value (NPV) of 94.9%, 93.9%, 94.6&, and 99%, respectively. Although the zone that under ROC of PCT level at 24 h had been 0.76 (% CI: 0.66–0.85), p = 0.001 confirmed the strong relevance of PCT in predicting in-hospital death rate at the perfect cutoff point of >0.15, where PCT level sensitivity, clarity, PPV, and NPV became 66.7%, 74.3%, 32.9%, and 92.2%, respectively (Fig. 1, Table 3). Furthermore, the ROC curve for PCT level 48/72 h was 0.89 (% CI: 0.85–0.93), p0.001, indicating that PCT has strong important predictive validity in estimating in-hospital deaths with sensitivity, precision, PPV, and NPV of 82.1%, 82.0%, 46.3%, and 0%, respectively. The area under the ROC of the PCT level at 5 days was 0.87 (% CI: 0.81–0.92), p0.001, indicating that PCT had significant predictive value in estimating in-hospital morbidity at the ideal cutoff point of >0.15, with.

**Fig. 2:**
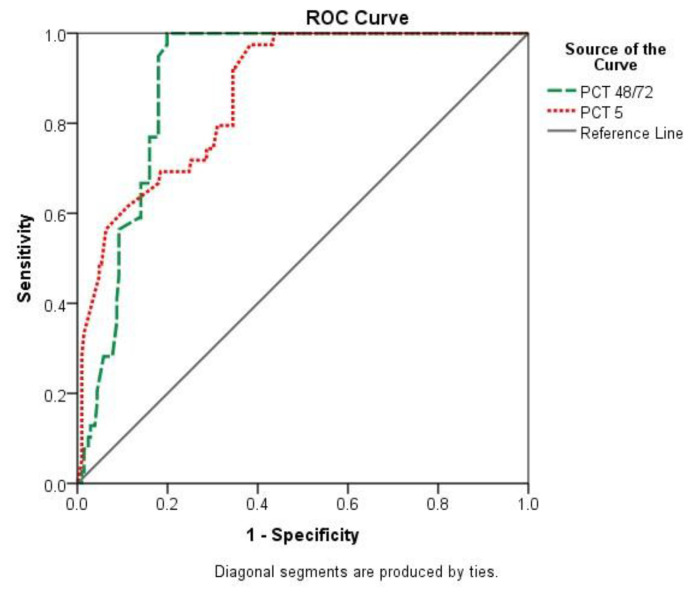
Comparison of predictive validity of PCT level on 48/72 h and day-5 to estimate patient’s in-hospital poor outcome. PCT 48/72 h AUC = 0.89 (95% CI: 0.85–0.93), p < 0.001 (High predictive validity). PCT 5 days AUC = 0.87 (95% CI: 0.81–0.92), p < 0.001 (High predictive validity).

**Fig. 3:**
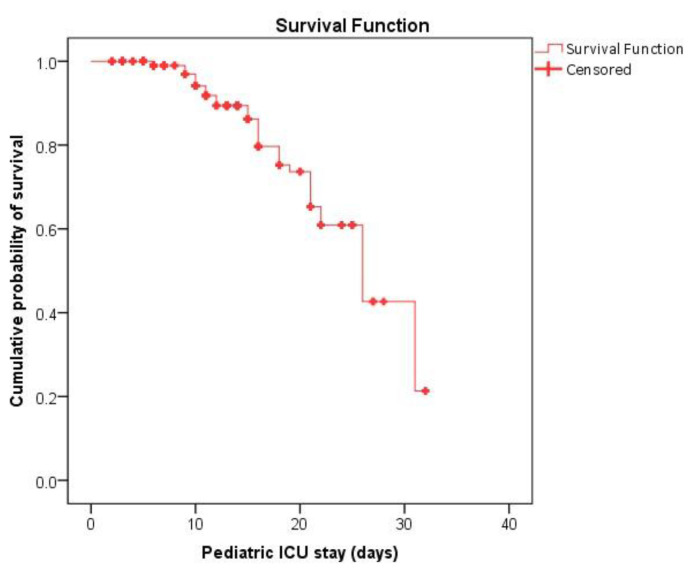
Survival analysis of pediatric patients in pediatric ICU. The mean survival time in PICU was 24.6 (95% CI: 22.8–26.3) days, highest probability of mortalities (90% or above) within first 15 days.

**Fig. 4:**
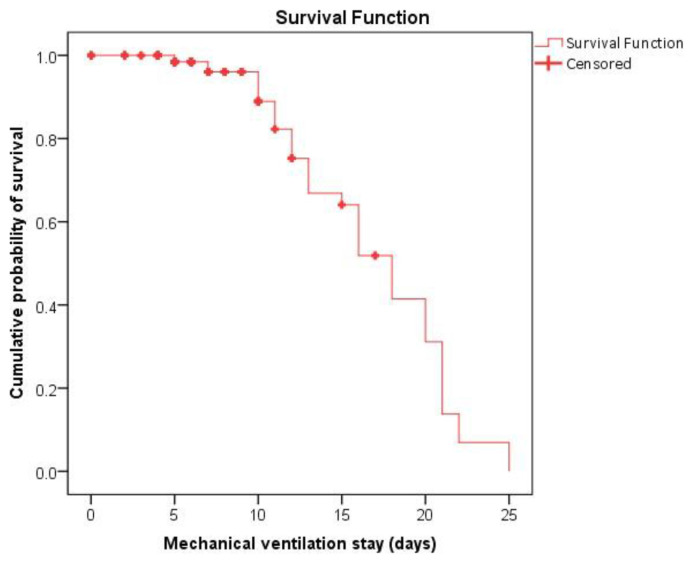
Survival analysis of pediatric patients in mechanical ventilation: The mean survival time on mechanical ventilator was 16.8 (95% CI: 15.4–18.2) days, highest probability of mortalities (95% or above) within first 7–8 days.

74.4% sensitivity, 71.4% specificity, 33.5% PPV, and 93.7% NPV, respectively (Fig. 2, Table 4). Inside the PICU, the average survival time was 24.6 (% CI: 22.8–26.3) days, with the largest mortality rate (90% or more) occurring within just 15 days (Fig. 3). The average duration of mechanical ventilation was 16.8 (95% CI: 15.4–18.2) days, with the highest mortality rate (95% or higher) occurring within 7–8 days (Fig. 4).

## 4. Discussion

Following postoperative infection, there were 39 (15.9%) deaths, of whom 22/39 (56.4%) were detected as septic, which would be higher than all that published in a local study [8], and also more than the mortality risk described in a systematic study [10], with the highest death rate of 11% [11]. In a Turkish report, PCT was found to be more accurate and relevant than C-reactive protein (CRP) and white blood cells in estimating morbidity in PICU patients, with an area under the ROC of 0.838 (0.711–0.966) and an 81.8 percent and 80.8 percent predictive accuracy at the better cutoff point of 6.38 ng/ml, respectively [6], which itself is consistent with the results of PCT at 48/72. Furthermore, the absolute sensitivity of the PCT level at 24 h and 74.3 percent, respectively, as well as the area under the ROC = 0.76 (% CI: 0.66–0.85), the appropriate cutting value > 0.15, were all significantly lower in the study. Similarly, Deng et al. [7] showed that the area under the ROC at entry for such a prediction of mortality was 0.76 in research based on neurontraumatic adult patients. In the frequency band of organ failure scores obtained (AUC 0.64 to 0.75 vs. AUC 0.53 to 0.65), our findings were considerably higher than those obtained by Masson et al. [12], who noticed preseason to have been an important biomarker than PCT, especially in relation to PCT with physical and physiological morbidity measurement in sepsis. Another study [13] reported an AUC of 0.805 in patients who required follow-up after recurrent trauma. In our research, students with poorer outcomes had significantly higher mean levels of biomarkers, such as PRISM III level, PCT at 24 h, PCT 48–72 h, and PCT at day-5 (p < 0.001), which matched the data of all studies in the literature review [14–16]. Repeated PCT measures for the first week following pediatric liver transplantation were not beneficial in identifying patients with bacterial infections, although serum PCT may well be efficient for the first week [17]. The research suggests that when seen as one element of a clinical puzzle, procalcitonin would provide critical information and that it is more effective when the interpreting physician understands how values are influenced by the various clinical circumstances given in this article [5]. Our work is a first in that it proved the predictive value of PCT level as a simple and quick method for estimating in-hospital outcome in post-surgical trauma cases that are prone to developing sepsis or other bacterial infections. Nonetheless, there isn’t a single lot of literature comparing the usage of PCT level in PICU patients with the optimal cutoff point for predicting in-hospital outcomes. According to our research, the average life expectancy of patients in the ICU was 25 days, with just a 90% chance of death during the first 15 days, but the average survival time on mechanical ventilation was 17 days, with a 95% chance of death within the first 7–8 days, according to our research. However, because there were only three deaths, they were unable to differentiate earlier organ dysfunction in order to further investigate predictive utility linked to outcome. Sole-Rebalta et al. sought to report a 5-day average stay in the PICU and a total of 14.5 days in the hospital [18]. There has only been one technique for comparing and defining it as the normal cutoff for evaluating the severity of effects, despite the fact that there are multiple PCT level cutoff points at different intervals. Another weakness of this study is its observational approach, which makes it impossible to show that PCT levels at certain points were utilized to identify the severity of an in-hospital outcome. Instead, a future cross-sectional investigation to see if raised PCT levels are as a result of the early planning or due to the use of non-invasive blood markers should be conducted.

## 5. Conclusion

The plasma procalcitonin concentration, as a single scientific marker, is remarkably accurate in assessing the in-hospital results of pediatric patients with post-surgical trauma, enabling the earlier therapeutic strategy to be initiated as a potentially life-saving intervention. During a prolonged hospitalization, a mixed approach based on PTC level and other clinical biomarkers could always be built for checking prescribed medications as well as converting to quality healthcare. The role of PCT level also can be investigated in prospective assessment studies limiting to comparable post-surgical trauma groups.

## Figures and Tables

**Table 1:** Association of demographic and clinical characteristics with sepsis and in-hospital outcome.

Variables	(n = 245)	Expired (n = 39)	Survived (n = 206)	Sig.
Gender				
Male	144 (58.8)	25 (64.1)	119 (57.8)	0.461
Female	101 (41.2)	14 (35.9)	87 (42.2)	
Etiology				
Neuro surgery	127 (51.8)	21 (53.8)	106 (51.5)	0.038
Pediatric surgery	78 (31.8)	9 (23.1)	69 (33.5)	
ENT & Ophthalmology	31 (12.7)	6 (15.4)	25 (12.1)	
Others	9 (3.6)	3 (7.7)^a^	6 (2.9)	
Ventilation				
Yes	235 (95.9)	39 (100)	196 (95.2)	0.371
No	10 (4.1)	0 (0)	10 (4.8)	
Blood culture				
Yes	179 (73.1)	24 (61.5)*	42 (20.4)	0.000
No	66 (26.9)	15 (38.5)	164 (79.6)	
Comorbidity				
Yes	88 (35.9)	25 (64.1)*	63 (30.6)	0.000
No	157 (64.1)	14 (35.9)	143 (69.4)	

a Shows highly significant proportion at 5% level of significance.

**Table 2:** Association of demographic and clinical characteristics with sepsis and in-hospital outcome.

Variables	Mean ± S.D	Expired (n = 39)	Survived (n = 206)	Sig.
Age (years)	5.27 ± 4.20	4.06 ± 3.92	5.50 ± 4.23	0.020
Weight (kg)	23.3 ± 11.4	16.7 ± 11.4	24.4 ± 11.0	0.000
PICU (days)	11.9 ± 7.18	16.3 ± 6.45	11.1 ± 7.02	0.000
ISS	5.76 ± 3.33	6.38 ± 4.70	5.64 ± 3.00	0.765
PRISM III	6.90 ± 5.41	17.4 ± 5.47	4.91 ± 2.11	0.000
PCT 24	0.43 ± 0.73	1.14 ± 0.95	0.30 ± 0.59	0.000
PCT 48/72	1.46 ± 2.48	4.15 ± 2.45	0.96 ± 2.13	0.000
PCT 5	0.54 ± 0.88	1.54 ± 1.14	0.35 ± 0.67	0.000
Ventilation days	7.51 ± 4.73	14.8 ± 5.76	6.14 ± 2.93	0.000
No. of inotropes	1.14 ± 1.11	2.00 ± 1.15	0.98 ± 1.02	0.000

**Table 3:** Cutoff points for predictive validity of PRISIM III, PCT 24 h, PCT 48/72 h and PCT 5 days in to estimate patient’s in-hospital poor outcome.

Cutoff	PRISM III (%)				Cutoff	PCT level 24 h (%)			
points	Sen.	Spe.	PPV	NPV	points	Sen.	Spe.	PPV	NPV
>6.5	100	60.7	32.5	100	>0.08	74.4	53.9	23.4	91.8
>7.5	100	74.8	42.9	100	>0.09	66.7	59.2	23.6	90.4
>8.5	97.4	87.4	59.4	99.4	>0.10	66.7	63.6	25.7	91.0
>9.5	94.9	93.9	94.6	99.0	>0.15	66.7	74.3	32.9	92.2
>11.0	89.7	99.5	97.1	98.1	>0.30	64.1	79.1	36.7	92.1
>13.0	87.2	99.5	97.1	97.6	>0.45	64.1	82.5	40.9	92.4
>15.0	82.1	100	100	96.7	>0.75	61.5	94.0	66.0	92.8

Sn: Sensitivity, Sp: Specificity, Prevalence (p = 15.9%) of in-hospital mortality.

**Table 4:** Cutoff points for predictive validity of PCT 48/72 h and PCT 5 days in to estimate patient’s in-hospital poor outcome.

Cutoff	PCT level 48/72 h (%)				Cutoff	PCT level 5 days (%)			
points	Sen.	Spe.	PPV	NPV	points	Sen.	Spe.	PPV	NPV
>1.00	92.3	82.0	49.2	98.2	>0.10	79.5	67.0	31.3	94.5
>1.15	84.6	82.0	47.1	96.6	>0.15	74.4	71.4	33.0	93.7
>1.35	82.1	82.0	46.3	96.0	>0.20	71.8	74.8	35.0	93.3
>2.00	76.9	82.0	44.7	94.9	>1.00	69.2	81.6	41.5	93.3
>2.80	66.7	85.9	47.2	93.2	>1.20	66.7	82.0	41.2	92.3
>3.00	60.0	85.9	44.6	91.9	>1.40	56.4	93.7	62.7	91.8
>3.50	56.0	91.1	44.5	91.9	>1.70	48.7	95.9	69.2	90.1

Sn: Sensitivity, Sp: Specificity, Prevalence (p = 15.9%) of in-hospital mortality.
